# Molecular signature of immunological mechanism behind impaired endometrial receptivity in polycystic ovarian syndrome

**DOI:** 10.20945/2359-3997000000476

**Published:** 2022-05-12

**Authors:** Fatemehsadat Amjadi, Zahra Zandieh, Mehdi Mehdizadeh, Marziyeh Ajdary, Azin Aghamajidi, Ehsan Raoufi, Reza Aflatoonian

**Affiliations:** 1 Iran University of Medical Science Akbarabadi Hospital Akbarabadi IVF clinic Tehran Iran Akbarabadi IVF clinic, Akbarabadi Hospital, Iran University of Medical Science, Tehran, Iran; 2 Iran University of Medical Sciences Department of Anatomical Sciences Tehran Iran Department of Anatomical Sciences, Iran University of Medical Sciences, Tehran, Iran; 3 Iran University of Medical Sciences Reproductive Sciences and Technology Research Center Tehran Iran Reproductive Sciences and Technology Research Center, Iran University of Medical Sciences, Tehran, Iran; 4 Iran University of Medical Sciences Endometriosis Research Center Tehran Iran Endometriosis Research Center, Iran University of Medical Sciences, Tehran, Iran; 5 Iran University of Medical Sciences School of Medicine Department of Immunology Tehran Iran Department of Immunology, School of Medicine, Iran University of Medical Sciences, Tehran, Iran; 6 Iran University of Medical Sciences School of Allied Medicine Department of Medical Biotechnology Tehran Iran Department of Medical Biotechnology, School of Allied Medicine, Iran University of Medical Sciences, Tehran, Iran; 7 Bioluence Biopharmaceutical Company Department of Vaccines and Immunotherapeutics Tehran Iran Department of Vaccines and Immunotherapeutics, Bioluence Biopharmaceutical Company, Tehran, Iran; 8 Royan Institute for Reproductive Biomedicine Department of Endocrinology and Female Infertility at Reproductive Biomedicine Research Center Tehran Iran Department of Endocrinology and Female Infertility at Reproductive Biomedicine Research Center, Royan Institute for Reproductive Biomedicine, ACECR, Tehran, Iran

**Keywords:** Endometrial receptivity, PCOS, QPCR array

## Abstract

**Objective:**

Despite the treatment of anovulation, infertility is still one of the main complications in PCOS women during reproductive age, which appears to be mainly due to impaired uterine receptivity. This study investigated the transcriptome profiles of endometrium in PCOS patients and healthy fertile individuals as the control group.

**Material and methods:**

Total mRNA was extracted from endometrial tissues of PCOS patients (n = 12) and healthy fertile individuals (n = 10) during the luteal phase. After cDNA synthesis, PCR array was performed using Human Female Infertility RT² Profiler PCR Array kit (Qiagen, Cat. No: PAHS-164Z) for evaluating expression of 84 genes contributing to the female infertility.

**Results:**

PCR Array data analysis identified significantly greater expression of *CSF, IL11, IL15, IL1r1, IL1b, TNF, LIF, TNFRSF10B, TGFβ, C3, ITGA4 (Cd49d), SPP1, and Calca* in PCOS women than in controls (P < 0.05). However, the expression of *LIFR, C2, CD55, CFD, CALCA, LAM1, LAMC2, MMP2, MMP7, MMP9, ESR, SELL, ITGB3,* and *VCAM1* was significantly lower in PCOS group than in controls (P < 0.05). The results revealed dysregulation of immune-inflammatory molecules, complement activation and downregulation of *IGF-I* as well as adhesion molecules in PCOS group.

**Conclusion:**

The findings of this study indicated some potential causes of reduced receptivity of endometrium thus compromising the fertility in PCOS patients.

## INTRODUCTION

Polycystic ovary syndrome (PCOS) is one of the most common endocrinopathies in women of reproductive age. PCOS approximately affects 4%-21% of women worldwide which varies depending on the criteria used for diagnosis (1). PCOS is commonly characterized by oligo-ovulation or anovulation, menstrual cycle abnormalities, ovarian polycystic morphology and endocrine problems, such as hyperandrogenism, hyperinsulinemia, and insulin resistance (IR), resulting in female infertility (2,3). PCOS patients are usually infertile, mainly due to the ovulation failure (4). Although anovulation can be treated by assisted reproductive techniques, pregnancy rates still remain low and high abortion rates can be observed in these patients (5,6). Several studies have indicated that decrease in uterine receptivity may be implicated in the infertility and implantation failure of the PCOS women (7,8). Previous investigations have suggested some protein networks regulate endometrial receptivity and coordinate interaction between endometrium and an embryo. Changes in these networks is responsible for the implantation failure and infertility (9). The underlying mechanisms of implantation failure in PCOS patients are still unclear (10).

Few studies have previously compared endometrium from PCOS patients to healthy fertile women in order to find potential markers for implantation failure in these patients (11). It was reported that downregulation of some endometrial molecules is involved in the adverse reproductive outcomes in PCOS patients such as avb3 integrin, *HOXA-10*, *HOXA-11*, and IGF binding protein 1 (*IGFBP-1*) (12,13). The overexpression of estrogen receptor and resistance to progesterone have also been shown in PCOS patients which may contribute to the impaired decidualization (14,15).

Our previous work revealed that the endometrium of PCOS patients bear a specific proteome signature distinctive of healthy fertile women. These differentially expressed proteins mostly participate in apoptosis, inflammatory, and immunological responses as well as cytoskeleton organization (16). However, none of previous investigations have confirmed a significant biomarker for uterine receptivity defects in PCOS. Thus, further detailed studies are required to elucidate which molecules or signaling pathways are altered in the endometrium of PCOS patients especially in the window of implantation.

In recent years, PCR array techniques can easily and reliably evaluate the expression of a specific panel of genes involved in a pathway with the features of a microarray analysis as well as sensitivity and specificity of real-time PCR (17).

This investigation employed Human Female Infertility RT^2^ Profiler PCR Array kit to compare the transcriptome profiles of endometrium in PCOS patients and healthy fertile women as the control group. It was worthwhile to identify the dysregulation of genes and pathways that may be affect endometrial receptivity and subduing the fertility in these patients.

## MATERIAL AND METHODS

### Sample collection

This study was approved by Institutional Review Boards of the Royan Institute (EC/92/1074) and all participants provided written informed consent. Endometrial biopsies were collected from infertile women with PCOS (n = 12) and healthy fertile controls (n = 10) seven days after the LH surge (LH+7). In this observational study, STROBE checklist was used for data collection and reporting (18).

**Figure 1:**
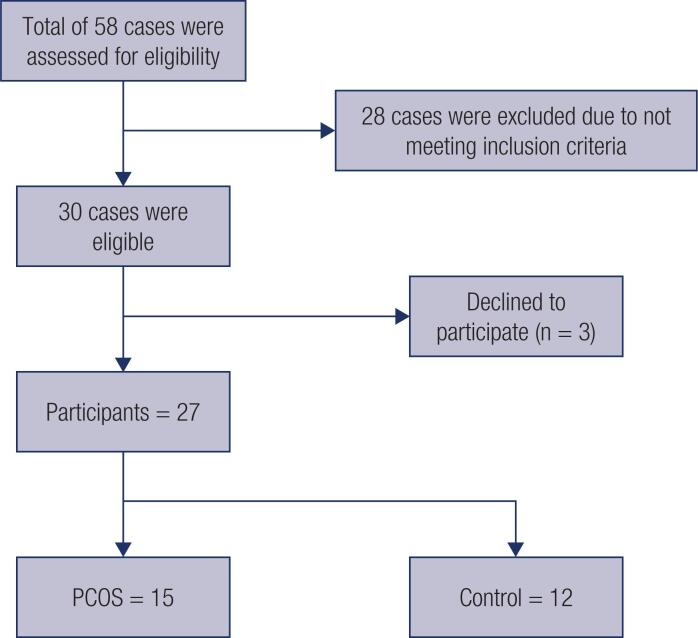
Participant flow. Participant flow chart and drop-out.

According to the following formula, the number of patients with PCOS was 15, of whom only 12 were willing to cooperate (19) (Figure 1).

For PCOS group, LH monitoring was initiated using urinary kit with intervals of every two days, 10-12 days after patients’ spontaneous menstrual bleeding. In addition to daily urine testing for LH, serum progesterone concentration was measured on the day of endometrial sampling, and in each case, ovulation was confirmed by serial transvaginal ultrasonography starting from approximately days 10-12 of the spontaneous menstrual cycle. Histological examination ensured that samples were taken during the mid-luteal phase. Monitoring ovulation in the PCOS patients took longer time than controls (3- 4 months vs. 1 month). PCOS was diagnosed according to the Rotterdam 2004 ESHRE/ASRM-Sponsored PCOS Consensus Workshop Group as observation of polycystic ovaries on the ultrasound scanning, the presence of oligo/anovulation, as well as clinical and/or biochemical signs of hyperandrogenism. Healthy controls had regular menstrual periods, normal FSH, LH, and estradiol concentrations on the day 3 of their menstrual cycle and had given birth to at least one child. The exclusion criteria were any uterine disease, endometrial hyperplasia, endometriosis, diseases related to the excess secretion of androgens, hypertension, and diabetes mellitus. Participants did not use any intrauterine device for contraception and not received hormonal therapy three months prior to the sample collection. The demographic characteristics are reported in Table 1. The tissue samples were taken by a Pipelle catheter under sterile conditions at Royan Institute as previously described by Amjadi and cols. (20). Specimens were placed into liquid nitrogen to snap freeze and then stored until RNA extraction.

**Supplementary Table 1:** Demographic characteristics of polycystic ovary syndrome and control subjects

Variable	PCOS n = 12	Control n = 10	*P value*
Age (Years [mean] ± SD)	27.31 ± 4.06	27.79 ± 3.89	NS
BMI (kg/m²)	27.42 ± 3.99	26.21 ± 4.19	NS
FSH (mU/mL)	8.12 ± 0.37	6.28 ± 1.90	NS
LH (mU/mL)	7.95 ± 3.96	4.00 ± 3.37	NS
LH/FSH ratio	0.98 ± 0.70	0.63 ± 0.33	0.03
Testosterone	2.65 ± 0.73	1.34 ± 0.53	0.04
AMH (ng/mL)	7.53 ± 5.18	2.48 ± 1.96	0.001
Progesterone	0.97 ± 0.26	0.77 ± 0.32	0.04
Cycle characteristic Interval between menstruation(days)	112.5 ± 21.98	29 ± 1.47	0.001

BMI: body mass index; NS: not statistically significant.

### RNA extraction and cDNA synthesis

RNA was extracted and purified using RNeasy mini kit (Qiagen, Cat. No: 73304) following the manufacturer's guidance and instructions. The concentration and purity of the RNA was checked using Nanodrop 2000 spectrophotometer (Thermoscientific). The total concentration of isolated RNAs and integrity was evaluated by the Picodrop system (Model; PICOPET01, UK). The first strand cDNA was synthesized using RT2 first strand kit (Qiagen, Cat. No: 330404).

### PCR-Array

PCR array was carried out by StepOnePlus^TM^ real time PCR system (ABI) using Human Female Infertility RT^2^ Profiler PCR Array kit (Qiagen, Cat. No: PAHS-164Z) with RT2 SYBR green ROX qPCR mastermix (Qiagen, Cat. No: 330502). The PCR-array kit contains five reference genes including *RPLP0*, *HPRT1*, *ACTB*, *B2M*, and *GAPDH*, whose data were analyzed by Norm-Finder algorithms. Based on the result, it was determined that ‘one’ is the optimum number of control genes and GAPDH ranked first for normalization based on our samples and genes of interest. Afterwards, it was also used for qPCR. The expression of 84 genes contributing to the female infertility relative to the GAPDH levels as reference gene was estimated via 2^−ΔΔCt^ formula. Each experiment was performed in triplicate.

### Statistical analysis

Normally and non-normally – distributed data were analyzed using independent sample T-test. Data in the text, tables, and figures are reported as means ± SD. All data were analyzed using Prism 8.0.2 software (GraphPad Software) and P < 0.05 was considered statistically significant. False discovery rate (FDR) was used for multi-comparison analysis.

## RESULTS

**Supplementary Figure 2:**
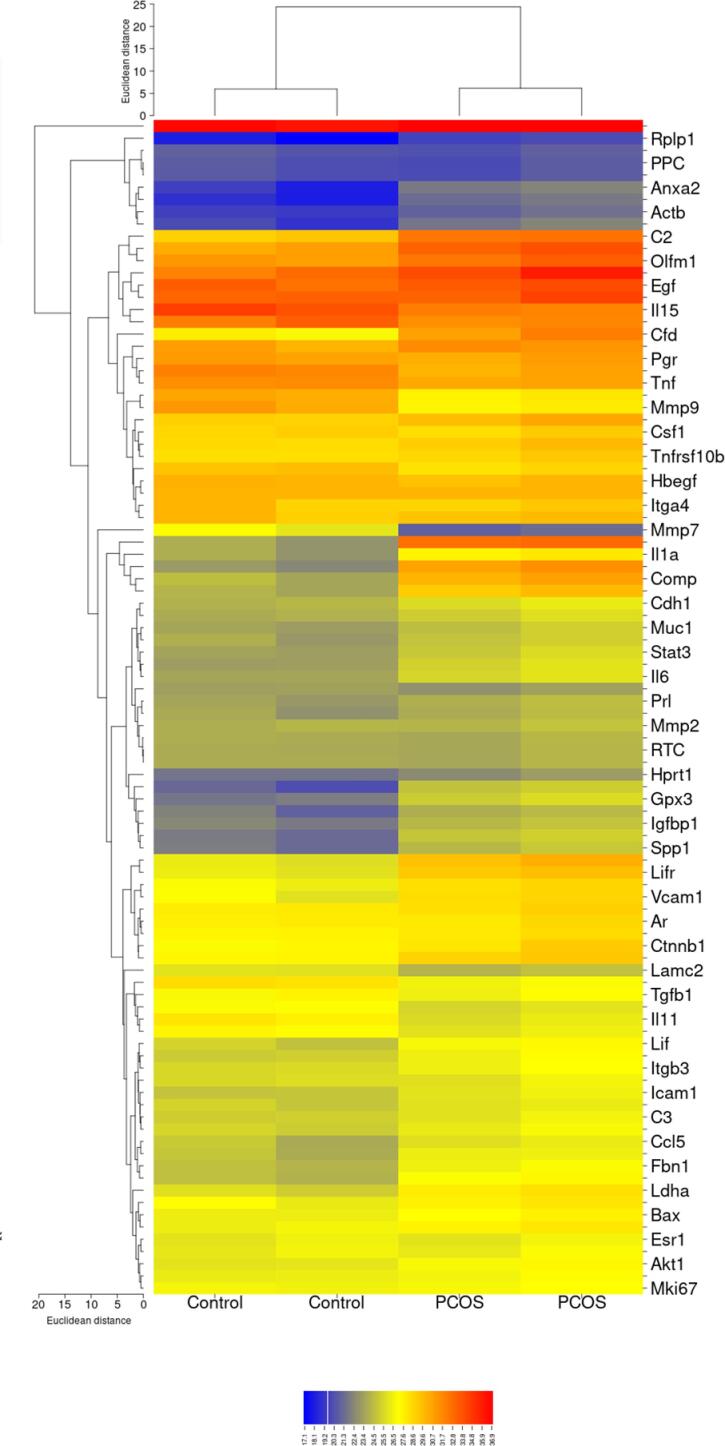
Analysis of integrated gene expression obtained from RT2 PCR Array (SABiosciences, Hilden, Germany). Gene expression data from the Control and PCOS were determined using hierarchical cluster analysis. Columns and rows represent examples of each group and gene, respectively. Each cell in the matrix represents the level of expression of a gene in the groups. High and low levels of gene expression are shown in blue and red, respectively.

We analyzed global gene expression of PCR-array profile containing a set of primers targeted for genes related to the female infertility pathway utilized to evaluate the alteration of these gene between endometrial tissue in PCOS and healthy fertile women. Significant changes (P < 0.05) in this pathway between the two groups, PCOS and control, are shown in Supplementary Figure 2.

**Figure 2:**
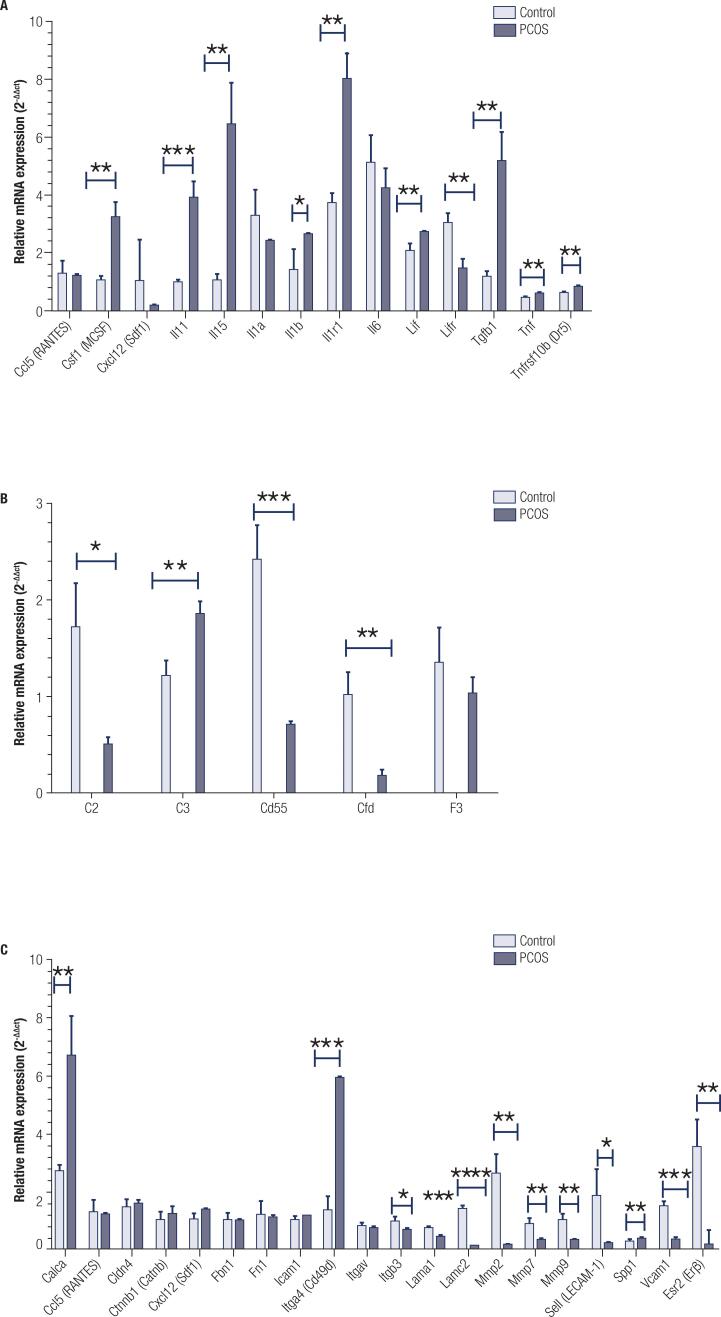
The relative mRNA level of cytokines (**A**), coagulation pathway (**B**), leukocyte migration pathway (**C**) in PCOS and normal groups. Data are the mean ± SD. * P < 0.05, ** P < 0 .01, *** P < 0.001 and ***P < 0.0001. * The comparison was made between PCOS group *vs.* Control groups.

PCR Array data analysis identified significantly greater expression of cytokine and cytokine receptors including *CSF1, IL11, IL15, IL1r1, IL1b, LIF, TNF, TNFRSF10B*, and *TGFβ* in PCOS women than in controls (P < 0.05). However, the expression of *CALCA* and *LIFR* was significantly lower in PCOS group than in controls (P < 0.05) (Figure 2 A).

The relative expression of complement system including *C2*, *CD55* and *CFD* was significantly lower in PCOS women than in controls (P < 0.05). However, the expression of *C3* was significantly greater in PCOS group than in controls (P < 0.05) (Figure 2 B).

PCR Array data analysis identified significantly lower expression of adhesion molecules and leukocyte migration pathway including *Lam1*, *Lamc2*, *MMP2*, *MMP7*, *MMP9*, *Sell*, *ESR*, *Itgb3*, and *VCAM1* in PCOS women than in controls (P < 0.05). On the other hand, the expression of *ITGA4* (*Cd49d*), *SPP1* and *Calca* was significantly greater in PCOS group than in controls (P < 0.05) (Figure 2 C).

## DISCUSSION

Our data indicated that the endometrium differs in PCOS women when compared to healthy individuals and PCOS contributes to dysregulation of endometrial genes expression. The comparative results of PCR array of Human Female Infertility genes between healthy fertile women and PCOS patients revealed a novel immunopathological cause of implantation failure. Type 2 immune response dominancy results in embryo implantation as a natural graft, while the type 1 responses results in inflammation which may lead to implantation failure (21).

**Figure 3:**
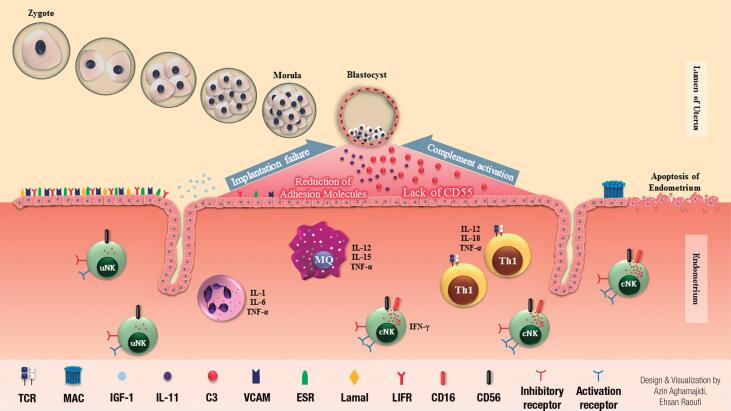
Immunopathogenesis of polycystic ovary syndrome. Implantation is the most fundamental process of a prosperous pregnancy in which adhesion molecules have an important role. The depletion of these adherent molecules causes a lack of blastocyst attachment to the uterus. Activation of immune responses, local inflammation of the uterus and imbalances in inflammatory and regulatory cytokines is one of the pathogenesis of infertility, probably. In PCOS patients, immune responses shift to Th1 which conducted to local inflammation and secretion of inflammatory cytokines including *IL-1B, IL-6, IL-12, IL-18, TNF-*α and *IFN-*γ. Following this inflammatory condition, *C3* molecule production by endometrial cells was increased which lead to complement activation due to a significant decrease in the *CD55* (*DAF*) regulatory molecule. Invasion of the membrane attack complex (MAC) to the endometrium, results in apoptosis, and endometrium destruction and blastocyst loss.

**Figure 4:**
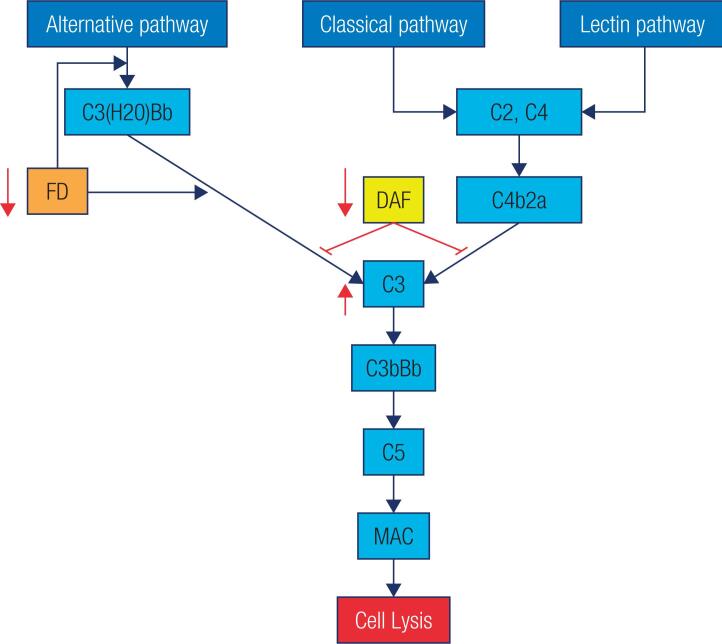
Complement pathway in PCOS patients. Increasing of complement components and regulatory proteins reduction simultaneously will trigger complement cascade activation and cell death. Red up and down arrows indicate increment and decrement of transcription level, respectively (p value ≤ 0.05). FD: complement factor D; DAF: decay accelerating factor (*CD55*); MAC: membrane attack complex.

According to the results, endometrial type 1 cytokines including *IL-1, IL-6, IL-11*, and *TNF-*α increased in PCOS group compared to controls. Interleukin 11 (*IL-11*), a multifunctional cytokine, has a critical role in successful implantation. Sever*al studies have indicated that upregulation of IL-11* is associated with inflammation. Although moderate increased inflammation occurs during implantation process, excessive levels of inflammatory factors lead to endometrial defectiveness. In PCOS patients, overproduction of *IL-11* by endometrial stromal cells may exacerbate the *C3* component amplifying the complement activation which may impair implantation process (22,23) (Figure 3). Complement activation is one of the immune defenses in inflammatory conditions which can be activated through different routes, and may lead to cellular damage (Figure 4). The complement cascade is activated through increased production of *C3* by endometrial cells, which results in MAC formation. Cell death occurred following the penetration of MAC into the membrane of endometrial cells and apoptosis induction (Figure 3). Thus, as chronic inflammation plays a crucial role in PCOS pathogenesis, loss of equilibrium between pro- and anti-inflammatory molecules during blastocyst implantation may contribute to infertility associated with this disease.

*IL-15*, as a pleiotropic cytokine, is involved in the production of T-helper1 (Th1) cells and proinflammatory cytokines as well as promoting proliferation and activation of T cells plus natural killer cells (24). Elsewhere, it was shown that *IL-15* increases in follicular fluid and serum samples of the PCOS patients, which may directly and/or indirectly contribute to implantation failure (25). In line with these results, the present study indicated the higher expression of *IL-15* in the endometrial tissues of the PCOS patients compared to the controls.

Previous data mining and review studies have presented *CD55* and *CFD* as putative biomarker of endometrial receptivity (26–30). In this study, the relative expression of complement system including *CD55* and *CFD* was significantly lower in PCOS women than in controls. Many studies have reported the upregulation of complement-regulatory molecules during the secretory phase of menstrual cycle, and they may also provide protective role for the embryo (27,31,32); our results indicated reduction of these factors in endometrial tissue of PCOs patients. In addition, adipsin (*CFD*) plays an influential and different role in embryo implantation as a prerequisite for production of oviduct-derived embryotrophic factor-3 (*ETF-3*) (33,34), which encourage embryo development (35,36). Hence, upregulation of adipsin in endometrium during the secretory phase in human may help the embryo during the implantation (37), but according to our results, this molecule is downregulated in endometrium of the PCOS patients.

On the other hand, matrix metalloproteinases (*MMPs*) have an important role in extracellular matrix (ECM) degradation during the implantation process. Studies have shown *MMP-2* and *MMP-9* are the main triggers of blastocyst implantation (38). *MMP7* is also generally expressed in endometrial epithelial cells and plays a significant role in the implantation process and endometrial receptivity (39,40). Our data revealed that the increased *MMPs* expression in the luteal phase of healthy women which may promote physiological apoptotic pathways moderately, beneficial for embryo implantation while endometrial *MMPs* expression was decreased in PCOS patients. This would lead to the decline of invasion capability of blastocyst and influence endometrial receptivity. One of the differentially expressed genes in PCOS patients is insulin-like growth factor-I (*IGF-I*). It has been suggested successful embryo implantation is a result of the *IGF*/*IGFBP* interaction which affects the inflammation balance (41). *IGF-I* has an intensive contribution to endometrial proliferation and differentiation. It has been upregulated during the early secretory phase and may have some role in preparing the endometrium for embryo implantation. Also, growth hormone activation promotes proliferation and vascularization of human endometrial cells in the *IGF-I*-mediated direction. This pathway upregulates the vascular endothelial growth factor (*VEGF*) and integrin beta 3 as a receptivity-related gene (42). *IGF-I* potentially regulates angiogenesis and integrin activation through the FAK signaling pathway. Furthermore, as animal model studies have shown, the *IGF-I* level was elevated in response to steroid hormone stimulation. The higher level of *IGF-I* plays a critical role in vascular permeability, decidualization, and upregulation of implantation markers (43,44). Our results indicated low level of endometrial *IGF-I* in PCOS women compared to healthy fertile controls which may result in implantation failure in these patients. Since ovarian stimulation protocols during ART cycles develop molecular alterations of the endometrium, genetic examination of endometrium in PCOS patients in ART cycles is needed. This study was limited by the number of subjects, and as such functional studies with a larger sample size are required to specifically assess the altered pathways discussed in this study.

In conclusion, this study demonstrated the altered molecular pathways in the endometrium of PCOS patients and supports a novel pathway which may cause decreased endometrial receptivity in PCOS. The imbalance of immune-inflammatory processes, including up-regulation of proinflammatory cytokines, changing in complement system, and diminished *IGF-I* expression as well as adhesion molecules may explain the molecular mechanistic details underlying endometrial aberrations and subsequently infertility in PCOS.
